# A Review on Anti-Inflammatory Activity of Phenylpropanoids Found in Essential Oils

**DOI:** 10.3390/molecules19021459

**Published:** 2014-01-27

**Authors:** Rita de Cássia da Silveira e Sá, Luciana Nalone Andrade, Rafael dos Reis Barreto de Oliveira, Damião Pergentino de Sousa

**Affiliations:** 1 Departamento de Fisiologia e Patologia, Universidade Federal da Paraíba, CP 5009, João Pessoa CEP 58051-970, PB, Brazil; 2 Departamento de Farmácia, Universidade Federal de Sergipe, São Cristóvão-SE, CEP 49100-000, SE, Brazil; 3 Departamento de Ciências Farmacêuticas, Universidade Federal da Paraíba, CP 5009, João Pessoa, CEP 58051-970, PB, Brazil

**Keywords:** phenylpropanoids, essential oils, natural products, medicinal plants, anti-inflammatory activity, inflammation, cytokines, immunomodulatory activity, asthma, allergy

## Abstract

The search for alternative drugs capable of disrupting the inflammatory process has become an important issue in scientific research, especially with reference to the use of natural substances and the reduction of undesirable side effects. Essential oils represent an important source of such substances, since their active constituents often exhibit an array of pharmacological properties, including anti-inflammatory activity. This review presents an overview of the anti-inflammatory action exerted by phenylpropanoids from essential oils and discusses possible mechanisms of action involved in the anti-inflammatory response, assessed through specific experimental models.

## 1. Introduction

Essential oils are natural volatile compounds that exhibit strong odors and are produced as secondary metabolites by aromatic plants [1]. They comprise complex mixtures of substances present in quite different concentrations, such as terpene and phenylpropanoid constituents. Historically, they have been used for various medicinal purposes, ranging from skin problems to cancer treatment and are known for their antimicrobial, anti-inflammatory, sedative and analgesic properties [2].

**Figure 1:**
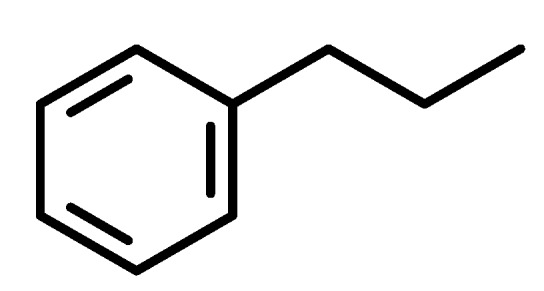
Phenylpropane skeleton.

Phenylpropanoids (Figure 1) are a large group of organic compounds produced by plants for protection against infections, ultraviolet irradiation, wounding and herbivores. They are synthesized from the amino acid phenylalanine, that is converted into cinnamic acid. Reduction of the carboxylic acid group present in the cinnamic acid yields an aldehyde (e.g., cinnamaldehyde) and further reduction produces monolignols such as phenylpropenes (e.g., eugenol and safrole). Natural and synthetic phenylpropanoids are under current medicinal use for their pharmacological properties [3,4]. 

Inflammation is a biological response to noxious stimuli such as pathogens that cause tissue and cell damage [5]. It is considered a protective measure taken by the organism to remove harmful stimuli and to begin the healing process. It is classified as either acute or chronic, depending on whether it involves a short response or a prolonged one, respectively [6]. The acute inflammatory response is initiated by plasma and leukocytes infiltration to the site of injury or infection [7]. It can be triggered by receptors of the innate immune system, for example the toll-like receptors (TLRs) [8]. In the first steps of infection, resident macrophages and mast cells release inflammatory mediators, such as cytokines (e.g., interleukin-1β (IL-1β), IL-6, IL-12, and the chemokine IL-8), tumor necrosis factors (e.g., TNF-α and TNF-β), interferons (e.g., IFN-γ), eicosanoids (e.g., prostaglandins and leukotrienes) and vasoactive amines (e.g., histamine) [7]. These mediators exert complex regulatory roles in the inflammatory process in order to restore tissue homeostasis.

Generally, cytokine production is induced by bacterial endotoxin or lipopolysaccharide (LPS) that activate TLRs (e.g., TRL-4) on the cell surface, initiating a series of intracellular events. The induction of innate immune and inflammatory responses by pathogens and non-microbial endogenous molecules involves the participation of TRL4 that act via two major signaling pathways (*i.e.*, MyD88- and TIR-domain-containing adapter-inducing interferon-β (TRIF)-dependent pathway). TRL4 is a transmembrane receptor and a binding site for LPS. One of the first steps in the TRL4 signaling pathway triggered by LPS binding involves oligomerization of the receptor followed by interaction with the adaptor molecules MyD88 and TRIF [9], leading to activation of nuclear transcription factor kappa-B (NF-κB), a ubiquitous rapid response factor involved in immune and inflammatory reactions that triggers the release of IL-1, IL-6 and TNF-α [9]. In particular, IL-1 and TNF may stimulate additional inflammatory pathways resulting in eicosanoid and nitric oxide (NO) production, induction of all adhesion molecules, and further cytokine production [10,11].

Chronic inflammation, on the other hand, is a dysregulated response to persistent noxious stimuli and seems to be related to tissue malfunction. This prolonged inflammatory condition is associated with a large number of chronic human disorders, including cancer, allergy, arthritis, atherosclerosis and autoimmune diseases [7].

In the past decades, the therapeutic potential of essential oils and their constituents has been the target of researchers in the pursuit of novel drugs of plant origin, particularly those exhibiting anti-inflammatory action, to be used in the prevention or treatment of diseases [12]. With the intent to provide information on this subject, this work presents a review of the anti-inflammatory effects of various phenylpropanoids that have been found to inhibit tissue inflammation *in vivo* and to modulate molecular pathways involved in inflammatory responses triggered by pro-inflammatory stimuli *in vitro*.

The present study was carried out based on the literature review of phenylpropanoids with anti-inflammatory activity from essential oils. Chemical structures and names of bioactive compounds as well as the corresponding references are provided (Table 1). The phenylpropanoids presented in this review were selected with reference to the effects shown in specific experimental models for evaluation of the anti-inflammatory activity and/or by complementary studies aimed to elucidate the mechanisms of action.

**Table 1:** Essential oil phenylpropanoids with anti-inflammatory activity.

Compound	Experimental protocol	Anti-inflammatory activity and/or mechanism	Animal/cell line tested	Reference
2′-hydroxycinnamaldehyde (2-propenal, 3-(2-hydroxyphenyl)-cinnamaldehyde) 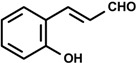	LPS-induced NO Production	Inhibitory of NO production	RAW 264.7 macrophage	[13]
	NF-kB-luciferase activity and NF-kB DNA binding activity	Suppression of luciferase activity		
	LPS-induced NF-kB activation and degradation of IkB	Inhibition of the production of nitrate, COX-2 and TNF-α		
Cinnamaldehyde [(2*E*)-3-phenylprop-2-enal] 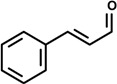	10 day-treatment (mixed on food)	Suppression of NF-κB activation	Fischer 344 rat	[14]
		Inactivation of IKK		
		Upregulation of IKBα and IKBβ		
		Inhibition of ERK and p38MAPK pathway		
	LPS-activated macrophage	Inhibition of NO and PGE_2_ production	RAW 264.7 macrophage	[15]
	LPS- or LTA-stimulated macrophage	Inhibition of IL-1β and TNF-α	Murine J774A-1 macrophage	[16]
		Suppression of pro-IL-1β production	Murine J774A-1 macrophage	
		Reduced release of ROS		
		Inhibition of phosphorylation of ERK 1/2 and JNK 1/2		
	OVA-sensitization	Increased IL-2, IL-4 and IL-10 production	Balb/c mice	[17]
	Cytokine-induced monocyte/human endothelial cell interaction	Suppression of VCAM-1 and ICAM-1	Human endothelial cell	[18]
		Suppression of NF-κB		
	LPS-activated BV2 microglia	Inhibition of NO production	BV2 microglia	[19]
		Decreases the expression of IL-1β, IL-6, and TNF-α		
		Reduced mRNA levels of iNOS		
	LPS-activated macrophage	Inhibiton of NF-κB and IRF3 activation	RAW 264.7 macrophage	[20]
	IL-1β-activation of COX-2 and PGE_2_	Reduction of COX-2 activity and PGE_2_ production	Rat cerebral microvascular endothelial cell	[21]
	LPS-induction of NO production	Reduction of NO release	J774 macrophage	[22]
	LPS-induction of COX-2 expression	Suppression of COX-2 expression		
	Carrageenan-induced paw edema	Reduction of paw edema	Rat	
Cinnamyl acetate 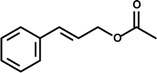	LPS-activated macrophage	Inhibition of NO and PGE_2_ production	RAW 264.7 macrophage	[15]
Cinnamic acid [(*E*)-3-phenylprop-2-enoic acid] 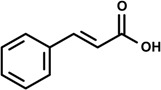	LPS-induction NO production	Inhibition of NO production	RAW 264.7 macrophage	[23]
Safrole (4-Allyl-1,2-methylenedioxybenzene) 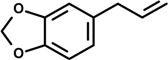	14 day-oral treatment	LPS inhibition of spleen cell suspension	Balb/c mice	[24]
		Suppression of response to B-cell mitogen, T-cell mitogen, and LPS		
	Splenocyte culture	Inhibition of response to LPS		
Dillapiole 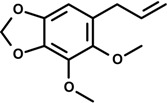	Carrageenan-induced paw edema	Inhibition of paw edema	Rat	[25]
Dihydrodillapiole 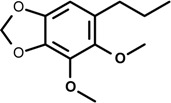				
Eugenol (4-allyl-2-methoxyphenol) 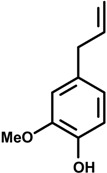	Croton oil-induced edema	Inhibition of edema	Mice	[26]
	Tooth pulp microsomes and homogenates	Inhibition of PG and leukotriene production	Rat tooth pulp homogenates	[27]
	Leukocyte		Human leukocytes	
	Kidney medulla		Rabbit and bovine kidney medulla	
	IL-1β-stimulated gingival fibroblast	Proinflammatory mediator in gingivitis	Gingival fibroblasts	[28]
	Platelet aggregation	Suppression of AA and PAF-induced platelet aggregation	Human platelet	[29]
	*In vivo* effects on AA and PAF-induced shock	Prevented the lethal effects of intravenous PAF or AA in a dose-dependent fashion	Rabbits	
	Carrageenan-induced paw edema	Reduction of edema	Rat	
	Platelet aggregation induced by AA	Inhibition of AA metabolism and thromboxane A_2_	Rat	[30]
Methyleugenol (4-allyl-1,2-dimethoxybenzene) 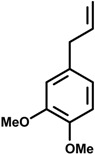	Cerebral ischemic model	Inhibition of NO and iNOS production	Rat	[31]
		Down-regulation of IL-1β, IL-6 and TNF-α		
		Increased expression of IL-10 and TGF-β		
Myristicin (5-allyl-3-methoxy-1,2-methylenedioxybenzene) 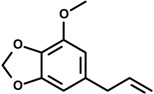	Double-stranded RNA-stimulated macrophage	Suppression of NO, IL-6, IL-10, GM-CSF, MIP-1α, MIP-1β, LIF and calcium production	RAW 264.7 macrophage	[32]
Elimicin (5-allyl-1,2,3-trimethoxybenzene) 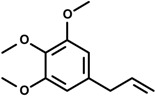	Basophil leukemia cells	Inhibition of 5-LOX	Rat	[33]
Asarone (1,2,4-trimethoxy-5-[(*E*)-prop-1-enyl]benzene) 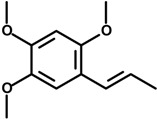	COX- enzymes inhibitory assay	Inhibition of COX-I and COX-II enzymes	Rat	[34]
Anethole [1-methoxy-4-(1-propenyl)benzene] 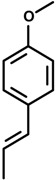	Carrageenan and persistent inflammation induced by Complete Freund’s Adjuvant	Suppressed paw edema	Mice	[35]
	Ear edema induced by croton oil	Inhibited the activity of myeloperoxidase	Mouse	[36]
	Carrageenan-induced pleurisy	Inhibiting the levels of NO and PGE_2_	Rat	
	LPS-induced acute lung injury	Reduced total protein concentrations	BALB/c mice	[37]
		Reduced numbers of inflammatory cells, such as neutrophils and macrophages		
		Reduced the production of inflammatory mediators matrix metalloproteinase-9 (MMP-9), TNF-α and NO		
Phenethyl alcohol 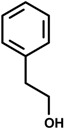	*In vitro* on the contraction of isolated tracheal smooth muscle	Prevented histamine-induced bronchoconstriction	Tracheal smooth muscle in guinea pig	[38]

The selection of essential oil constituents in the database were related to various terms, including essential oils and phenylpropanoids, as well as names of representative compounds of these chemical groups, further refining with keywords like asthma, antiasthmatic effect, allergy, antiallergic effect, inflammation, anti-inflammatory, immune response, lymphocytes, cytokines, immunoglobulins or immunoregulatory. The search was performed in the scientific literature databases and Chemical Abstracts and covers until November 2013.

## 2. Results and Discussion

### 2.1. Cinnamaldehyde (trans-Cinnamaldehyde, 2′-hydroxycinnamaldehyde) and Cinnamic Acid

*Cinnamomum cassia* has been widely used for treating dyspepsia, gastritis, and inflammatory diseases. The anti-inflammatory activity of derivatives synthesized from various cinnamic acid based on the 2’-hydroxycinnamaldehyde (HCA) isolated from the bark *C. cassia* Blume was investigated to compare their NO production and NF-kB activity from Raw 264.7 cell. The results showed that HCA, among the derivatives, most significantly inhibited lipopolysaccharide (LPS)-induced NO production and NF-kB transcriptional activity in a dose-dependent manner with an IC_50_ value of 8 and 22 mM, respectively [13].

Cinnamaldehyde is a constituent of essential oils known to exert anti-cancer, antifungal, cytotoxic mutagenic/anti-mutagenic and anti-inflammatory effects. It is the major component of cinnamon bark oil used as a flavoring agent in foodstuffs such as ice cream. An increasing number of studies have described the anti-inflammatory activity of cinnamaldehyde elicited by different signaling pathways that regulate the anti-inflammatory responses. For instance, the assessment of the anti-inflammatory effect of cinnamaldehyde and its role in the regulation of age-related alterations in signal transduction pathways evidenced the participation of the molecular regulation of NF-κB activation by cinnamaldehyde via the redox-related NF-κB-inducing kinase/IkB kinase (NIK/IKK) and mitogen activated protein kinase (MAPK) pathways through decreased oxidative stress [14].

In this context, NF-κB has been shown to be activated by various stimuli such as oxidative stress, and besides activating pro-inflammatory genes through diverse kinases, it also modulates cellular signaling mechanisms involved in inflammation induced by oxidative stress during aging [39,40,41]. In a study by Kim and collaborators [14], male Fischer 344 rats were fed a diet containing 2 or 6 mg/kg of cinnamaldehyde for 10 days. The results showed that cinnamaldehyde inhibited age-related oxidative stress and suppressed age-related NF-κB activation that upregulated NF-κB targeting genes, inducible nitric oxide synthase (iNOS) and ciclo-oxygenase-2 (COX-2). Besides the NIK/IKK pathway, the signal transduction extracellular-signal-regulated kinase (ERK) and p38 MAPK pathways are also involved in the inhibition of NF-κB, however, during aging, the increased degradation of the cellular inhibitory protein of the NF-κB transcription factor known as IκB by IKK leads to increased binding activity of NF-κB [14]. Cinnamaldehyde, on the other hand, inhibited this activity by inactivating IKK thereby resulting in the upregulation of nuclear factor of kappa light polypeptide gene enhancer in B-cells inhibitor, alpha (IκBα) and IκBβ. Additionally, cinnamaldehyde suppressed NF-κB activation by inhibiting the ERK and p38 MAPK pathways, suggesting that the antioxidative effect and the restoration of redox balance were related to the anti-inflammatory activity displayed by this compound [14].

*Cinnamomum osmophloeum* Kaneh (Lauraceae) is a tree that grows in Taiwan whose components extracted from the leaf essential oil have been reported to exhibit many biological effects such as anti-microbial, anti-fungi and anti-inflammatory [14,42,43]. It is also used to treat influenza and the common cold [44]. Indeed, the essential oil of *C. osmophloeum* twigs was shown to be effective on NO and prostaglandin E2 (PGE_2_) production in LPS-activated Raw 264.7 macrophages. Among the constituents of the twig essential oil obtained from *C. osmophloeum* are the phenylpropanoids *trans*-cinnamaldehyde and cinnamyl acetate. *In vitro* studies designed to evaluate NO and PGE_2_ production in LPS-stimulated RAW 264.7 cells showed that, at the concentration of 10 μg/mL, cinnamaldehyde (59.9%) exerted stronger inhibitory effect on NO production than the twig essential oil (46.2%), while cinnamyl acetate (48.1%) displayed similar action to that of the twig essential oil. At the same dose level, cinnamaldehyde inhibited PGE_2_ production by 65%, and indomethacin (positive control), a non-steroidal anti-inflammatory drug, exhibited 98% inhibition [15].

Chao and collaborators [16] also showed the *in vitro* anti-inflammatory activity of cinnamaldehyde (24–80 μM) obtained from the essential oil of *C. osmophloeum* leaves. The following effects were observed: inhibition of IL-1β and TNF-α production in LPS- or lipoteichoic acid (LTA)-stimulated murine J774A-1 macrophages and in LPS-stimulated human blood monocytes derived primary macrophages and THP-1 monocytes; suppression of pro-IL-1β production in LPS- or LTA-stimulated human THP-1 monocytes; reduced release of reactive oxygen species (ROS) from LPS-stimulated J774A-1 macrophages; and inhibition of phosphorylation of ERK 1/2 and c-jun N-terminal kinase 1/2 (JNK 1/2) induced by LPS. As these subtypes of MAPKs have been implicated in the regulation of cytokines gene expression, such as TNF and IL-1, as evidenced in culture human and murine macrophages [45], it is believed that cinnamaldehyde inhibitory action on cytokines synthesis could be related to the reduction of ERK 1/2 and JNK 1/2 activation in J774A-1 macrophages stimulated by LPS. In this case, these findings give further evidence of the anti-inflammatory activity played by cinnamaldehyde involving the participation of MAPKs and their regulatory role in immune responses, suggesting its potential pharmaceutical employment as an immunomodulatory agent [16].

*In vivo* cytokine modulatory effects of cinnamaldehyde as well as the leaf essential oil from *C. osmophloeum* were investigated in a murine model by use of ovalbumin (OVA)-primed Balb/c mice treated daily for four weeks. The leaf essential oil did not exert any significant cytokine-modulatory effects, but cinnamaldehyde-treated animals (1 mg/kg) exhibited significantly increased serum levels of IL-2, IL-4 and IL-10, but not IFN-γ [17]. The significance of these results lies in the fact that these cytokines exert counteracting roles in inflammatory events: IL-2, for instance, mediates macrophage activation, helping to protect hosts from infections, and stimulates the development of type 1 T helper cells (Th1) [46], while IL-4 increases production of Th2 cells, and IL-10, secreted by Th2 cells, inhibits IFN-γ synthesis by Th1 cells, and reduces pro-inflammatory cytokine production [47].

**Figure 2:**
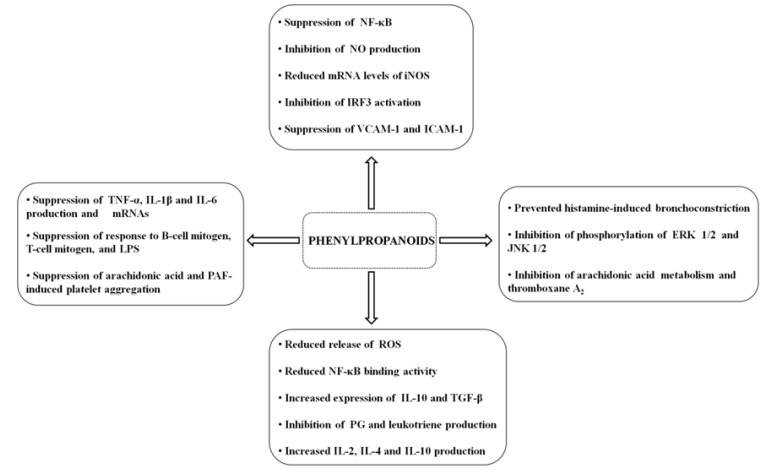
Possible mechanisms of action from phenylpropanoids anti-inflammatory activity.

*Cinnamomum cassia* (Nees & T.Nees) J.Presl (Lauraceae), popularly known as cinnamon, is a spice used in traditional medicine in the treatment of gastritis and also has pharmacological properties such as anti-inflammatory and anti-tumor activity [48,49]. The effect of *C. cassia* on atherogenesis has been assessed, providing further evidence of the anti-inflammatory action of this species. Atherogenesis is a chronic inflammatory process related to the accumulation of lipid-containing plaques on the innermost layer of the wall of an artery, which involves the production of adhesion molecules, such as vascular cell adhesion molecule-1 (VCAM-1) and intercellular cell adhesion molecule-1 (ICAM-1), followed by leukocyte attachment to endothelial cells [50,51]. Another important element taking part in this process is TNF-α which activates NF-κB, cytokines, and adhesion molecules inducer, hence contributing to the increase of the inflammatory response [52,53]. The study of the effects of cinnamaldehyde obtained from *C. cassia* and extracts of this species on cytokine-induced monocyte/human endothelial cell interactions showed that the adhesion of TNF-α-induced monocytes to endothelial cells were significantly inhibited by these components. The suppression of VCAM-1 and ICAM-1 was observed at the transcriptional level and NF-κB—the main downstream signal of VCAM-1 and ICAM-1—was also suppressed in TNF-α-treated endothelial cells. In short term treatment, the anti-inflammatory effects of cinnamaldehyde were displayed by interruption of the degradation of IκBα, while in long term treatment, the anti-inflammatory effects of cinnamaldehyde were via the induction of Nrf2-related genes, such as heme oxygenase-1 (HO-1), known to be associated with inhibition of TNF-α-induced ICAM-1 expression [18]. Additionally, cinnamaldehyde upregulated Nrf2 nuclear extensions, increased the activity of antioxidant response element (ARE)-luciferase, and upregulated another Nrf2-related gene, namely thioredoxin reductase-1. These findings indicate that cinnamaldehyde has two distinct mechanisms, activated by different treatment periods, to suppress TNF-induced signaling pathways [54] (Figure 2).

Neurodegenerative diseases (e.g., Alzheimer’s disease and Parkinson’s disease) involve neuroinflammatory responses mediated by microglia, which can be activated by endogenous and environmental stimuli such as LPS, leading to the activation of transcriptional factors such as NF-κB and activator protein 1 (AP-1). As a consequence, proinflammatory genes are upregulated, resulting in the release TNF-α, IL-1β, IL-6, NO and ROS [19,55]. In a study by Ho and collaborators [19], the anti-neuroinflammatory activity of cinnamon and cinnamaldehyde were investigated with respect to their ability to suppress NO, TNF-α, IL-1β, and IL-6 production, mRNA expression of iNOS, TNF-α, IL-1β, and IL-6, and DNA binding activity of NF-κB in LPS-activated BV2 microglia. After treatment with LPS, BV2 microglia exhibited increased NO production which was significantly inhibited by cinnamon ethanolic extract (65% at 50 μg/mL), while cinnamaldehyde inhibited NO production by 45.3%, 70.8% and 98% at 25, 50 and 100 μM, respectively. Cinnamon extract (50 μg/mL) and cinnamaldehyde (100 μM) reduced mRNA levels of iNOS, indicating that the inhibition of NO production was probably related to down-regulation of iNOS gene expression. The production of TNF-α, IL-1β, and IL-6 was increased in LPS-treated BV2 microglia, but cinnamon extract (50 μg/mL) significantly reduced their production by 65.7%, 85.0% and 71.0%, respectively, as well as mRNA levels of TNF-α (69.1%), IL-1β (89.5%), and IL-6 (59.5%). Cinnamaldehyde (100 μM) also suppressed TNF-α, IL-1β, and IL-6 (>50%) production and their respective mRNA levels, suggesting that cinnamon extract and cinnamaldehyde inhibited gene expression of these proinflammatory cytokines. LPS-treated BV2 microglia showed increased DNA binding activity of NF-κB, however both cinnamon extract and cinnamaldehyde reduced NF-κB binding activity by 80.9% and 77.2%, respectively. These findings indicate that cinnamon and cinnamaldehyde exhibited anti-neuroinflammatory activity, displaying potential therapeutic employment against neurodegenerative diseases.

Other studies have discussed the inhibition of NF-κB pathway by cinnamaldehyde and its interrelation with TLRs. As mentioned earlier, the activation of TLR4 is associated with the increase of the inflammatory response as it leads to the activation of pro-inflammatory elements such as cytokines, COX-2, NOS, NF-κB, interferon regulatory factor 3 (IRF3), a component that plays an important role in the innate immune system’s response to viral infection [56], and the expression of inflammatory mediators which may involve My88- and TRIF-dependent signaling pathways [57]. *In vitro* assays using RAW 264.7 macrophages showed that LPS-induced activation of NF-κB and IRF3 (TLR4 agonist) was inhibited by cinnamaldehyde, resulting in reduced expression of target genes such as COX-2 and IFN-β. Although cinnamaldehyde failed to suppress the activation of NF-κB or IRF3 induced by MyD88- and TRIF-depend pathways, the oligomerization of LPS-induced TLR4 was inhibited by cinnamaldehyde leading to the downregulation of NF-κB activation [20]. Moreover, cinnamaldehyde inhibited ligand-independent NF-κB activation induced by constitutively active TRL4 or wild-type TRL4. These findings suggest a different mechanism for the anti-inflammatory activity of cinnamaldehyde by targeting the oligomerization of TLR4 and not downstream signaling molecules, indicating that TRL4 and the signaling components may become interesting therapeutic targets for inflammation-related chronic diseases [20].

Guizho-Tang is a Traditional Chinese Medicine used in the treatment of pyretic conditions including colds and influenza [21]. In pathogenic infections, fever is a systemic response that may be triggered by LPS release from gram-negative bacteria resulting in the production of cytokines, such as IL-1β, which has been shown to increase the mRNA expression and activity of COX-2 and PGE_2_ production in rat cerebral microvascular endothelial cells (RCMEC) [21,58]. The effects of one of the main active compounds of Guizho-Tang, namely cinnamaldehyde, on the expression and activity of COX and PGE_2_ were assessed in RCMEC in a study by Guo and collaborators [21]. The data obtained corroborated the findings of Uracz and collaborators [58] by showing that IL-1β did induce mRNA expression and activity of COX-2 and PGE_2_ production in RCMEC, but did not alter significantly the mRNA expression and activity of COX-1. Cinnamaldehyde (200—800 μM), on the other hand, when added to cultured RCMEC, reduced IL-1β-induced COX-2 activity and IL-1β-induced PGE_2_ production, but did not alter significantly COX-2 mRNA and COX-1 activity. These findings indicate that cinnamaldehyde may play a role in Guizhi-Tang’s anti-pyretic activity by inhibiting IL-1β-induced PGE_2_ production, a well-known mediator of fever through inhibition of COX-2 activity [21,59].

*Ocotea quixos* (Lam.) Kosterm (Lauraceae) is a species native to Ecuador and Colombia that has a cinnamon-like aroma, and is used as a spice called ishpingo [60]. The essential oil extracted from this species has been shown to exert anti-thrombotic effects in mice by inhibiting platelet aggregation, clot retraction and vasoconstriction [61]. The *in vitro* investigation of *O. quixos* essential oil and two of its major constituents, *trans*-cinnamaldehyde and methyl cinnamate, showed that *O. quixos* essential oil and *trans*-cinnamaldehyde (1–10 μg/mL) significantly reduced LPS-induced NO release from J774 macrophages, while methyl cinnamate failed to inhibit NO production up to the highest concentration (10 μg/mL). Moreover, the oil and *trans*-cinnamaldehyde suppressed LPS-induced COX-2 expression and elevated production of cAMP induced by forskolin. *In vivo*, both the essential oil (30–100 mg/kg) and *trans*-cinnamaldehyde (10 mg/kg) exhibited anti-inflammatory action by reducing carrageenan-induced rat paw edema. Methyl cinnamate (30 mg/kg), on the other hand, did not suppress the anti-inflammatory effects induced by carrageenan. Also, all animals treated with indomethacin (10 mg/kg—positive control) exhibited ulcered gastric mucosa while *O. quixos* essential oil- and *trans*-cinnamaldehyde-treated rats did not show any gastric injury. In fact, the administration of *O. quixos* essential oil (100 mg/kg) and *trans*-cinnamaldehyde (10 mg/kg), but not methyl cinnamate (30 mg/kg), prevented gastric mucosa injuries after oral administration of 90% ethanol to rats. These findings indicate that *trans*-cinnamaldehyde exerts anti-inflammatory effect endowed with gastroprotective property [22].

Some information is available in the literature about the regulatory effect of cinnamaldehyde on monocytes and macrophages activation by LPS, treatment of monocytes with proaggregative antibodies and the use fibronectin, an extracellular matrix protein. Cinnamaldehyde acted as a strong regulator of monocyte/macrophages-mediated immune responses and inhibited NO production, the upregulation of surface levels of CD80 and CD69 (costimulatory molecules) and pattern recognition of TLR2 and complement receptor 3 (CR3). It also obstructed cell-cell adhesion induced by CD29 and CD4 activation, but did not interfere with cell-fibronectin adhesion. Further analysis using immunoblotting techniques indicated that the inhibitory effect of cinnamaldehyde was due to inhibition of phosphoinositide-dependent kinase 1 (PDK1) and NF-κB [62].

In a different approach, the effect of cinnamaldehyde on the lipoxygenase (LOX) pathway was assessed with respect to leukotrienes production (e.g., LTA4, LTC4, LTD4, LTE4 and LTB4). Their pro-inflammatory action includes increased vascular permeability, contraction of airway smooth muscle (LTD4 and LTE4), adherence of neutrophils to endothelial cells (LTB4), working as a chemoattractant, and stimulation of plasma exudation [63]. Among the experimental protocols employed to investigate the potential anti-inflammatory action of compounds, there is the indirect approach which involves measuring the suppressor effects on the LTB4 formation *in vitro*. In this context, cinnamaldehyde was reported to exert a strong inhibitory effect on the production of LTB4 [64].

In addition to *trans*-cinnamaldehyde, cinnamic acid is another phenylpropanoid found in the essential oil of plants such as *Panax ginseng* C.A. Meyer (Apiaceae), known in China and Korea as a respected herbal medicine used to maintain physical vitality [65]. An *in vitro* study showed that cinnamic acid significantly inhibited LPS-induced NO production in RAW 264.7 macrophages and effectively suppressed oxidative damages in the human neuroblastoma SH-SY5Y cells, suggesting that this compound contributes to the neuroprotective and anti-inflammatory actions of *P. ginseng* [23]. The inhibitory effect of cinnamic acid on the oxidative pathway was also evidenced in earlier studies, in which *in vitro* protocols using Guinea pig peritoneal macrophages showed that O2 generating response caused by N-formyl-Met-Leu-Phe (fMLP) was suppressed by cinnamic acid. It was suggested that the inhibitory activity displayed by this component was possibly related to reduced calcium mobilization since cinnamic acid also inhibited calcium mobilization elicited with fMLP [66].

### 2.2. Safrole, Dillapide, Dihydrodillapide, Eugenol and Methyleugenol

Safrole, a compound found in a wide variety of plants, is a mutagenic and carcinogenic agent [67]. *In vivo* and *in vitro* studies evaluated the effects of safrole administered orally to female Balb/c mice for 14 days (100, 200 and 400 mg/kg). On the fourth day, Ig-M antibody response to T-dependent antigen revealed that safrole inhibited sheep red blood cells at all dose levels, and LPS of spleen cell suspensions from treated animals. After 14 days of treatment, the assessment of mitogen-induced proliferation of splenocytes showed that the responses to B-cell mitogen, LPS, and T-cell mitogen concanavalin A (ConA) were significantly suppressed at a dose of 400 mg safrole/kg. Direct exposure to safrole in the splenocyte culture also produced inhibition of *in vitro* antibody response to LPS, and mitogen-induced lymphoproliferation (100, 200, 400 and 800 μM). Further analysis using splenocyte-hepatocyte coculture system was employed to evaluate the role of metabolic activation in safrole-induced suppression of *in vitro* antibody response. The inhibition of the response to LPS by safrole did not change when compared with the direct exposure to safrole in splenocytes culture. Moreover, the exposure to salicylamide, sulfotransferase inhibitor, and inorganic sulfate, sulfation cofactor to the splenocyte-hepatocyte coculture did not interfere with suppression of antibody response by this compound. These findings indicate that the immunosuppressive action exhibited by safrole is probably not related to the production of the reactive metabolites which are mediated in carcinogenesis by safrole [24].

In another study, the anti-inflammatory action of safrole, dillapiole and its analog dihydrodillapiole was evidenced in the carrageenan-induced rat paw edema model. These compounds were isolated from essential oil extracted from *Piper aduncum* L. (Piperaceae), a species known to exert protective action against phytopathogens (e.g., bacteria and fungi) and to have analgesic effect. Dillapiole and dihydrodillapiole significantly suppressed paw edema whereas safrole showed lower inhibitory action than indomethacin (positive control), indicating a possible use of dillapiole and dihydrodillapiole as compounds exhibiting anti-inflammatory action [25].

Eugenol is a compound known to display pharmacological properties that include hypothermic, antioxidant, anti-inflammatory and local anesthetic actions. In traditional medicinal, it is used against gastrointestinal disorders such as flatulence and chronic diarrhea [68]. Studies have shown that eugenol suppressed TNF signaling and COX-2 expression, which suggests its potential use as an anti-inflammatory agent [69]. In an earlier study, Rasheed and collaborators [70] reported that eugenol extracted from nutmeg oil suppressed the production of thromboxane B2 by washed platelets, giving further support to eugenol’s potential role as an anti-inflammatory agent.

The major mechanism of the anti-inflammatory action of non-steroidal anti-inflammatory drugs usually involves inhibition of PG production by selective inhibition of COX. Phenolic dental medications (e.g., phenol, *p*-chlorophenol, eugenol, *o*-cresol and guaiacol) are used as disinfectants in root canal treatments and in the treatment of pulpitis and dentine hyperalgesia. These medications have different effects on PG synthesis, which can be both stimulatory and inhibitory, depending on the concentration (phenol, *p*-chlorophenol, and *o*-cresol), small stimulatory and potent inhibitory effects (guaiacol), or only inhibitory effects (eugenol) [26]. *In vitro* studies in human leukocytes, bovine, rabbit and rat tooth pulp homogenates, and rabbit and bovine kidney medulla showed that eugenol and *m*-cresol inhibited PG and leukotriene biosyntheses, suggesting that their anti-inflammatory effect may involve the AA metabolism pathway [27].

A study by Dohi and collaborators [26] corroborated these findings as topically applied eugenol, *o*-cresol and guaiacol inhibited croton oil-induced edema formation in mice (doses: 0.2 and 0.5 mg per site 15 min before or 1.0 and 2.0 mg per site 60 min after croton oil application). Since *in vivo* models of edema induction are related to increased levels of PG synthesis, the results indicate possible involvement of suppression of PG production as the participating mechanism in the anti-inflammatory effect displayed by phenolic dental medications.

The effect of eugenol as an anti-inflammatory agent in dental treatment was investigated in IL-1β-stimulated gingival fibroblast and pulp cells through the evaluation of IL-8 production, which is often associated with inflammation, in particular as a proinflammatory mediator in gingivitis. Eugenol (5–500 μM) significantly increased IL-8 synthesis in gingival fibroblasts, but exhibited bi-modal effects on pulp cells as it produced modest stimulation at lower concentration (5 μM) and a significant suppressive effect at higher concentration (500 μM). Similar results were also observed in LPS-stimulated gingival fibroblasts and pulp cells, indicating that eugenol exerted anti-inflammatory activity on pulp cells but not fibroblasts [28].

In China, many traditional medicinal herbs—e.g., *Angelica sinensis* (Oliv.) Diels (Apiaceae), *Lonicera japonica* Thunb (Caprifoliaceae) and *Bupleurum chinense* (DC.) Franch. (Apiaceae)—are widely used due to the extensive biological actions exhibited by the essential oils and their active principles. Among the major active constituents present in many Chinese medicinal herbs, eugenol modulates the function of immune-responsive cells. It has been shown that eugenol (10 μM to 100 μM) triggered intracellular calcium mobilization in Jurkat T-cells and THP-1 monocytic cells and stimulated the active phosphorylation of the p 38 subgroup of MAPK (EC_50_ values for both were around 10–30 μM) [71]. The mobilization of intracellular calcium is one of the first steps that occur during activation of immune-responsive cells. As a consequence, calcium activates a cascade of kinases that, in turn, may lead to cell proliferation and increased cytokine production [72].

In a different context, the role of eugenol as an antiplatelet agent, a platelet-activating factor (PAF) and arachidonic acid (AA) inhibitor was investigated. PG and thromboxanes play important roles in the platelet aggregation process and in acute inflammation by stimulating the release of AA metabolites and PAF, a phospholipid that has been shown to aggregate human platelets, improve vascular permeability and increase contraction of bronchial smooth muscle [29,73]. Consequently, PAF is believed to work as a mediator of thrombosis and inflammation. Platelet activation by d (AA) leads to further liberation of AA that is then metabolized by LOX and COX pathways. One example of metabolization is the production of thromboxane A2 that leads to increased intracellular calcium levels by stimulating the hydrolysis of phosphatidylinositol-4,5-biphosphate [30,74]. The *in vitro* study on the effects of eugenol on human platelet aggregation, AA and PAF metabolism showed that eugenol suppressed AA and PAF-induced platelet aggregation (IC_50_ values: AA—31 ± 0.5 μM and PAF—0.7 ± 0.2 μM). Eugenol also inhibited the metabolism of AA via COX and LOX pathways and thromboxane A2 in a concentration dependent manner (30–120 μM). Pretreatment *in vivo* of rabbits with eugenol (50–100 mg/kg) prevented the lethal effects of intravenous PAF (11 μg/kg) or AA (2 mg/kg) in a dose-dependent fashion. The protective effects of eugenol in the rabbits, however, were more pronounced against PAF-induced mortality (100% protection). Furthermore, *in vivo* studies showed that pretreatment of rats with eugenol (25, 50 and 100 mg/kg) significantly reduced carrageenan-induced paw edema by 28%, 62% and 78%, respectively. Aspirin (used as positive control) at the dose levels of 150 and 300 mg/kg exhibited 25% and 73% reduction in edema volume, indicating that eugenol exerted more potent anti-inflammatory effect than aspirin in the paw edema model [29].

Corroborating these findings, Chen and collaborators [30] showed that eugenol and sodium eugenol acetate (a compound synthesized from eugenol by sodium acetate displacement of the 4-phenolic hydroxyl group) dose dependently (0.25, 0.5 and 1.0 mM) inhibited AA-induced thromboxane B2 and PGE_2_ formation. It also inhibited intracellular calcium increase caused by AA, collagen, ADP and adrenalin (1 mM). Moreover, the inhibitory effect of eugenol and safrole on leukotriene production was reported by Ahn and collaborators [75]. They suggested that the inhibitors can be used to treat a vast number of inflammatory diseases including asthma, allergic rhinitis, cystic fibrosis, rheumatoid arthritis and inflammatory intestine disease.

Liver injury caused by hepatotoxins usually involves damage to the centrilobular regions resulting in increased ROS production, lipid peroxidation and release of pro-inflammatory cytokines, such as TNF-α and IL-6, produced by non-parenchyma cells (e.g., Kupffer cells and hepatic stellate cells) [75,76]. In this context, eugenol was evaluated with respect to its ability to exert anti-inflammatory, antioxidant and DNA-protective role in thioacetamide-induced hepatic injury in rats. Eugenol significantly reduced liver damage, lipid peroxidation, protein oxidation, and inflammatory cytokines production, and improved antioxidant activity. It also prevented thioacetamide–induced DNA strand break and significantly inhibited the over-expression of COX-2 gene induced by thioacetamide [77].

Methyleugenol is a compound structurally similar to eugenol found in the essential oils of many plant species. It is popularly used as an anti-allergic [78] and anti-nociceptive agent [79]. Cerebral hypoxia/ischemia is a serious brain injury caused by many interrelated factors that includes increased intracellular calcium accumulation, oxidative stress and inflammation [31,80]. A study by Choi and collaborators [31] investigated the effect of methyleugenol in *in vivo* and *in vitro* cerebral ischemic models. After occlusion of the middle cerebral artery (for 1.5 h) followed by reperfusion (for 24 h), methyleugenol (applied shortly after reperfusion) effectively improved cerebral ischemic injury. Additionally, the following effects were observed: lower generation of superoxide in the ischemic brain and reduced intracellular oxidative stress caused by oxygen-glucose deprivation/re-oxygenation; increased superoxide dismutase and catalase activity; inhibition of NO production and iNOS protein; and down-regulation of pro-inflammatory cytokines synthesis (e.g., IL-1β, IL-6 and TNF-α) in the ischemic brain as well as in immunostimulated mixed glial cells. Methyleugenol also increased mRNA expression of anti-inflammatory cytokines such as IL-10 and TGF-β. These findings indicate that methyleugenol exerts a cytoprotective effect by reducing cerebral ischemic injury by suppression of oxidative injury and inflammation, suggesting a possible use in the treatment of ischemia inflammation related diseases [31].

### 2.3. Myristicin, Elemicin, Asarone, Anethole and Phenethyl Alcohol

Myristicin is a phenylpropanoid found in carrot, basil, cinnamon, sparsely and nutmeg (seed of *Myristica fragrans* Houtt., Myristicaceae). In traditional medicine, it is used in the treatment of diarrhea, stomach aches and anxiety [81]. It is also known to display anti-cholinergic, antibacterial and hepatoprotective properties [82]. An *in vitro* evaluation of the anti-inflammatory action of myristicin (10, 25, and 50 μM) on double-stranded RNA-stimulated RAW 264.7 mouse macrophages showed that the production of calcium, NO, IL-6, IL-10, interferon inducible protein-10, monocyte chemotactic protein (MCP)-1, MCP-3, granulocyte-macrophage colony-stimulating factor (GM-CSF), macrophage inflammatory protein (MIP)-1α, MIP-1β, and leukemia inhibitory factor (LIF, a member of IL-6 family) was significantly suppressed by this compound. These results indicate that myristicin displays anti-inflammatory activity by inhibiting various participants of the inflammatory response and suggests the involvement of the calcium pathway in this process. Pathogenic oxidative stress with infection results in increased intracellular calcium concentration leading to stimulation of calcium-dependent kinases and pro-inflammatory activation [83]. As myristicin decreased the levels of intracellular calcium, it is believed that the production of inflammatory mediator in macrophages is being down-regulated by myristicin via calcium pathway [32].

Further consideration indicated that myristicin could be useful in the treatment of various diseases. For instance, myristicin may play a regulatory role in chronic autoimmune diseases such as Crohn’s disease, rheumatoid arthritis and encephalomyelitis by inhibition of excessive production of IL-6, LIF and IL-10 [84]. The later, although known as an anti-inflammatory cytokine, participates in the pathobiological mechanism of autoimmune diseases such as lupus and encephalomyelitis [85]. Also, myristicin may attenuate lung inflammatory diseases (e.g., bronchial pneumonia and chronic asthma caused by viral infection), since it significantly inhibited the synthesis of PIC-induced MCP-1, MCP-3, GM-CSF, MIR-1α and MIP-1β, which are implicated in the inflammatory response of many lung diseases. Moreover, it might exert anti-inflammatory action against pathogenic infections caused by bacteria and viruses, since it inhibits NO production, which is thought to be a major pro-inflammatory mediator in these infections [32].

Elemicin is a constituent of the essential oils of nutmeg and mace and it is believed to be partially responsible for the hallucinogenic effects of nutmeg [86]. Studies on anti-allergic activity of various compounds extracted from the methanolic extract of *Asiasarum sieboldii* (Miq.) F. Maek (Aristolochiaceae) roots showed that the elemicin inhibited 5-LOX from rat basophil leukemia cells (RBL-1 cells)—cancerous cells commonly used in the study of allergy—indicating the possible role of elemicin as an anti-allergic agent [33]. This plant is from the phylum Magnoliophyta, class Magnoliopsida and order Piperales. In the AA pathway, 5-LOX converts essential fatty acids into leukotrienes, which are directly related to the expression of pathological symptoms in asthma. Therefore, 5-LOX inhibitors are important targets for pharmaceutical interest to be developed as asthma treatments [87,88].

Asarone is a phenylpropanoid found in related herbs. The anti-inflammatory action of asarone was demonstrated in cyclooxygenase (COX) enzyme inhibitory assays. The investigation of *Daucus carota* seed extracts resulted in the isolation and characterization of this compound, which showed 46.15% prostaglandin H endoperoxide synthase-I (COX-I) inhibitory activity and 64.39% prostaglandin H endoperoxide synthase-II (COX-II) inhibitory activity, both at 100 mg·mL^−1^ [34].

Anethole is a natural constituent of essential oils of many plants such as the spice star anise (*Illicium verum* Hook. f., Illiciacea) and is known to have antioxidant, antifungal, antibacterial, anesthetic and anti-inflammatory properties [89]. Two inflammatory pain models—acute inflammation induced by carrageenan and persistent inflammation induced by Complete Freund’s Adjuvant—showed that the oral treatment with anethole (125, 250 and 500 mg/kg) in mice suppressed paw edema, myeloperoxidase (MLP) activity, and decreased the levels of TNF-α, IL-1β and IL-17, indicating that anethole exert anti-inflammatory activity in acute and persistent inflammatory models [35]. The anti-inflammatory action of anethole was also investigated by Domiciano and collaborators [36] by use of the croton oil-induced ear edema and carrageenan-induced pleurisy models. The oral treatment with anethole (250 and 500 mg/kg) significantly reduced the volume of migrated leukocytes, and the levels NO and PGE_2_ in the inflammatory exudates; however, contrary to the findings of Ritter and collaborators [35], TNF-α and IL-1β levels were not significantly altered. In the ear edema, the oral treatment with anethole, but not the topical application, reduced exudate formation and MLP activity. Moreover, another experimental study using the LPS-induced acute lung injury model in BALB/c mice showed that the intraperitoneal administration of anethole (250 mg/kg) reduced total protein concentrations, number of inflammatory cells, such as neutrophils and macrophages, and the inflammatory mediators matrix metalloproteinase-9 (MMP-9), TNF-α and NO. Anethole also reduced LPS-induced histopathological changes and inhibited NF-κB activation by blocking IκB-α degradation. These findings give further evidence of the anti-inflammatory activity of anethole, suggesting its possible therapeutical employment in the treatment of inflammation in humans [37]. The bioactive compounds discussed in this review are oxygened and low polarity molecules. Most are esters, while others are alcohol, acids, aldehyde, and methylenedioxy or methoxylated compounds. Oxygenation occurs on the aromatic ring or the terminal carbon of the carbon chain. A comparison of the pharmacological effects is not appropriate because the experimental conditions and models are different. However, data from studies show that the phenylpropanoids exhibit anti-inflammatory activity via different mechanisms of action.

Finally, Wuhu decoction, a traditional Chinese formulation employed in the treatment of asthma, has several active components, including the phenylpropanoid phenethyl alcohol, which was investigated *in vitro* on the contraction of isolated tracheal smooth muscle in guinea pig. Phenethyl alcohol prevented histamine-induced bronchoconstriction, which indicates anti-asthmatic effect, corroborating the traditional use of this formulation as an anti-asthmatic agent [38].

## 3. Conclusions

Considerable attention is being given to the discovery of novel drugs capable of fighting inflammation, particularly those of plant origin. Essential oils and their active constituents, such as the phenylpropanoids, are a promising source of anti-inflammatory substances and the data presented in this review show the potential roles the phenylpropanoids can display in this field. The information available in the scientific literature indicates the participation of phenylpropanoids in different mechanisms of action related to the immunomodulation and suppressive action in the inflammatory response as disclosed in *in vitro* and *in vivo* experimental protocols. It is expected that further studies involving clinical trials will be carried out in order to ensure a safe use of these substances as a therapeutic agent against inflammatory diseases.

## Abbreviations

AA: Arachidonic acid
c-AMP: Cyclic adenosine monophosphate
COX-1: Cyclo-oxygenase-1
COX-2: Cyclo-oxygenase-2
ERK: Extracellular signal-regulated kinases
GM-CSF: Granulocyte-macrophage colony-stimulating factor
IC: Inhibitory concentration
ICAM-1: Intercellular Adhesion Molecule 1
IFN-γ: Interferon gama
IL1-β: Interleukin 1β
IL-6: Interleukin 6
IL-12: Interleukin 12
IRF3: Interferon regulatory transcription factor 3
LPS: Gram negative bacteria lipopolysaccharide
LOX: Lipoxygenase
LTB4: Leukotriene B4
LTC4: Leukotriene C4
LTD4: Leukotriene D4
LTE4: Leukotriene E4
MAPK: Mitogen-activated protein kinases
MCP-1: Monocyte chemoattractant protein-1
MCP-3: Monocyte chemoattractant protein-3
NF-Κb: fator nuclear kappa B
NO: Nitric oxide
NOS: Nitric oxide synthase
mRNA: Messenger RNA
PAF: Platelet-activating factor
PGE_2_: Prostaglandin E2
ROS: Reactive oxygen species
TNF-α: Tumor necrosis factor-alpha
TGF-β: Transforming growth factor-beta
